# Report on Certain Points Connected with the Histology of Minute Blood Vessels, to the Surgeon General of the U. S. Army, by Brevet Lieut. Col. J. J. Woodward, Assistant Surgeon U. S. Army, with Eleven Accompanying Photo-micrographic Illustrations

**Published:** 1870-11

**Authors:** 


### Editorial Department.

*Report on certain points connected with the Histology of Minute Blood Vessels, to the Surgeon General of the U. S. Army, by Brevet Lieut. Col.* J. J. WOODWARD, *Assistant Surgeon U. S. Army, with eleven accompanying Photo-micrographic Illustrations. From the War Department Surgeon General's Office.*

Our readers are already acquainted with the name, and with some of the previous efforts of the already distinguished author, who has had the honor, through the kindness of Surgeon General Barnes, of publishing this and other valuable reports to the professional world. Those who were present at the meetings of the American Association, in Washington, last June, will, doubtless, be familiar with the subject-matter of this report, as also with the excellence of the manner in which were presented to the Association a series of illuminated screen represesentations, being enlarged *fac-simile* reproductions of the microscopic appearances of certain histological preparations, which are deposited in the Microscopical section of the Army Medical Museum.

Surgeon Woodward, to whom the thanks of the Association were voted, has, by request, prepared duplicate sets of lantern slides, similar to those used before the Association, and these will be much desired by those who are interested in histological studies, and those who wish to present to students the results of late investigations in histology and surgical pathology.

Photographic copies on paper, from eleven negatives, selected from the number which have been prepared, are now issued from the Surgeon Generals' office, and these accompanying a brief, printed report, very concisely written, and from which we quote:

"Having recently been occupied in the critical examination of certain preparations, in the microscopical section of the Museum, illustrative of the minute anatomy of blood-vessels, I have thought that some of them threw so much light on certain points involved in the recent discussions with regard to the doctrine of inflammation, that a short account of them would be of interest, and might, perhaps, do good service in connection with the appreciation of the conflicting statements which have appeared in the Medical Journals since the publication of the paper of Dr. J. Conheim, on inflammation and suppuration. Perhaps the observation of Cohnheim must fairly be regarded as observations of the previous experiments of Dr. Augustus Waller, but they certainly produced an impression upon the medical world far beyond that made by the papers in the *Philosophical Magazine*, and more or less complete account of conclusions arrived at by the distinguished Berlin observer have continued to appear, from time to time, in both foreign and American Medical Journals, ever since the publication of his papers in 1867.

After I had perused Cohnheim's paper, I carefully repeated many of the experiments described. I received the impression from what I saw, that Cohnheim was a most conscientious observer, who had described as faithfully as possible the impressions made upon him. Certainly the results I obtained, by following his methods of producing inflammation in the cornea, and mesentery of frogs, could be described in his very language, without drawing upon the imagination. It is simply my desire to contribute what is in my power towards the important object of arriving at certainty with regard to the facts on which our future theories of inflammation are to rest.

Most of the preparations here referred to, are examples of the results attainable by staining the tissues with a dilute solution of nitrate of silver. This reagent has been employed for various histological purposes during the last ten years, and has attracted attention especially in connection with the cornea, the various forms of connective tissue, the ultimate branches of the lymphatics and the boundaries of the cells which constitute epithelial surfaces. If a dilute solution of nitrate of silver is brushed over a clean epithelial surface taken from a recently killed animal, or is injected into the blood vessels, and the tissue after washing with distilled water, is exposed for a short time to the action of sunlight, it will be found on microscopical examination that a brownish black precipitate of silver has been produced at the boundaries of the epithelial cells, while the cells themselves are comparatively but little stained, or if the manipulation has been carefully conducted, are not stained at all. For this purpose I have most frequently employed at the museum, a solution made by dissolving one part of crystallized nitrate of silver in four hundred parts of distilled water, but considerable variation on either side of this strength does not much modify the result provided the solution is well washed off before the tissue is exposed to the light. It is often found advantageous to combine the silver solution intended for injection, with a certain amount of gelatine, by which the blood vessels are kept handsomely distended and the beauty of the preparation is much increased.

The silver staining having been successfully accomplished, the nuclei are tinted preparably by the solution of carmine in borax, described by Thiersch in his work on epithelial cancer. It is prepared as follows: Four parts of borax are dissolved in fifty-six parts distilled water, and one part of carmine added to the solution; one volume of this fluid is mixed with two volumes of absolute alcohol, and after crystals have formed the mixture is filtered. The filtrate may be used for staining, but if the crystals of carmine and borax which remain on the filter are dissolved in a small quantity of distilled water, I find the solution thus obtained answers a still better purpose. The portion of tissue to be studied is soaked in this solution until it is colored deep red. It is afterwards treated with a saturated solution of oxalic acid in alcohol, by which all color is gradually removed, except from the nuclei. So soon as that is accomplished, the piece is to be carefully washed in alcohol, then soaked in absolute alcohol and finally mounted in a solution of dried Canada Balsam, in chloroform or benzole."

The various authors whose experiments have an important part in the history of the subject, are mentioned, and their writings referred to, while in the report may be found the full particulars in reference to the method of the preparation of the specimens. It may be here stated that by means of a camera arrangement attached to a microscope and with the help of the magnesium or calcium light, photographic negatives are obtained direct from the microscope and this process of obtaining photographs is called mico-photography. A full description of each of the photographs then follows, from which we mainly condense, as follows:

I. Photograph representing several venous radicals uniting to form a small vein in the muscular coat of the urinary bladder of a frog. Magnified 400 diameters and illuminated by magnesium lamp. The walls of the venous trunk and of those of its branches, which are in focus, are plainly seen to be formed of somewhat irregular epithelial cells, which vary in size, averaging 1-500th of an inch in length and 1-2200th in breadth. The boundary of each cell is indicated by a zigzag black line. In each of the cells which is accurately in focus, a smooth, oval nucleus, I-2800th of an inch in length is visible. Two kinds of nuclei belonging to the muscular coat of the bladder, are also to be here seen.

II. A small vein from another portion of same preparation. Magnified 1000 diameters, shows several blood corpuscles.

III. Stomata between the epithelial cells of a vein 1-50th of an inch in diameter in the mesentery of a frog, magnified 400 diameters These stomata are certain irregularly rounded forms, situated in the line of division between adjoining cells, and present an appearance similar to that of Wormian bones as they are situated in cranial sutures, and they vary in diameter from 1-10,000th to the 1-4000th of an inch in diameter.

IV. Stomata in a more minute vein than before, viz, 1-100th of an inch in diameter.

V. Stomata in a still more minute vein, viz., 1-1000th of an inch in diameter. Here several of the stomata present clear centres, while others are black and opaque throughout.

VI. A minute artery with part of the adjoining net-work of capillaries, from the muscular coat of the urinary bladder of the frog, magnified 400 diameters. The artery is 1-1700th of an inch in diameter. Its epithelial cells are longer in proportion to their width than those of the venous epithelium. The epithelial cells of the walls of the capillaries are also plainly shown. In the intervascular spaces the nuclei of the muscle and connective tissue appear as in the first photograph.

VII. A portion of the same magnified 1000 diameters.

VIII. Small artery in the mesentery of the frog, magnified 500 diameters. The artery is 1-280th of an inch in diameter, and is marked by both transverse and longitudinal silver lines. The former are exterior to the latter, and indicate the boundaries of the circular fibres of the muscular coat, the latter that of the cells of the epithelial coat.

IX. Epithelium of a capillary 1-2300ths of an inch in diameter in the muscular coat of the urinary bladder of the frog, magnified 1000 diameters. The author then remarks upon the certainty and accuracy with which the cell boundaries are mapped out by silver, carmine, etc., and says that the question as to whether the discoloration is in the cell wall or in the cement or matrix by which the adjacent cells are held together, is one which he does not now propose to enter; it is enough for the purposes of the present paper to say that the peripheries of the cells, or the substance just external to them, does exhibit a more intense reaction with the nitrate than the cell contents do, hence must differ more or less from these in composition. Next, as to the stomata, he says that they, almost invariably are found in the boundary lines between adjacent cells, the exceptions, probably, being explainable, and that their centres being sometimes transparent, and from that varying to opacity, may be due to their being occupied by fluids of variable composition. From his studies he is inclined to regard with favor the opinion that stomata are actual openings in the epithelial layers.

X. Silver staining of the epithelium of the frog's skin, magnified 400 diameters. The cells are hexagonal in form, and average in diameter 1-5000th of an inch. Stomata 1-5000th inch in diameter are shown, some of which have dark centres, some transparent. It is known that through the external epithelium of a frog's foot, a rapid transudation of liquid habitually occurs. That stomata are pores through which pass the white blood corpuscles of the blood, seems to me not difficult of understanding. Balogh's objection that the stomata are usually only one-third the diameter of the corpuscles themselves, may be obviated, even if we discard the supposition that the stomata are capable of distension. One who has seen the extraordinary modifications of form which these little masses of protoplasm undergo in their so called "amœboid movements," would readily credit their capability of passing through such apertures. As the amœboid movement does not occur in the white corpuscles while rolled along in the torrent of the circulation, but only when the movement of the blood is arrested more or less completely, the fact that large numbers of white corpuscles do not habitually pass through the vascular wall into the tissues will not militate against the notion of patulous orifices. That a passage of the white blood corpuscles through the vascular walls does actually occur, is shown by the next photograph.

XI. White corpuscles in various phases of the amœboid movement, in the external coat of a small vein of the muscular coat of the Stomach of a mare magnified 400 diameters. The preparation is one of a number of sections made from the stomach of a mare, dead of gastro-enteritis. In these sections which are stained and with carmine and mounted in Canada Balsam after the method before described, it was found that many of the small veins of the sub-peritonal connective tissues, and of the muscular coat were surrounded by white corpuscles fixed in all stages of amœboid movement. In a number of places where the sections pass transversely through the veins, the white corpuscles can be observed in the interior of the urine and in the vascular walls as well as in the adjacent tissue. The series of preparations give a satisfactory demonstration of the wanderings of the white corpuscles.

In conclusion, the author states that these studies as far as they go, confirm the statements of Cohnheim, but as to the doctrine of inflammation, and of the transformation of corpuscles into tissue, they rank as ingenious hypothesis not yet proved. The interesting and original manner in which the whole profession is now enabled to participate in the investigations of the foremost microscopists is sufficient apology for the extended notice here presented. Of the great excellence and superior merit of Surgeon Woodwards mico-photographs we wish our readers to be self constituted judges.

