# Errata

**Published:** 1870-12

**Authors:** 


ERRATA.—In the November number of the Journal, page 154, line 15, word 9, read "*photo-micography;*" page 156, line 12, word 9, read "*vein;*" same page, line 17, word 10, read "*hypotheses;*" same page, line 17, word 9, read "*photo-micographs.*"

